# Indirect ELISA based on Hendra and Nipah virus proteins for the detection of henipavirus specific antibodies in pigs

**DOI:** 10.1371/journal.pone.0194385

**Published:** 2018-04-30

**Authors:** Kerstin Fischer, Sandra Diederich, Greg Smith, Sven Reiche, Vinicius Pinho dos Reis, Eileen Stroh, Martin H. Groschup, Hana M. Weingartl, Anne Balkema-Buschmann

**Affiliations:** 1 Friedrich-Loeffler-Institut, Federal Research Institute for Animal Health, Institute of Novel and Emerging Infectious Diseases, Greifswald-Insel Riems, Germany; 2 Canadian Food Inspection Agency, National Centre for Foreign Animal Disease, Winnipeg, Canada; 3 Friedrich-Loeffler-Institut, Federal Research Institute for Animal Health, Department of Experimental Animal Facilities and Biorisk Management, Greifswald-Insel Riems, Germany; University of Texas Medical Branch at Galveston, UNITED STATES

## Abstract

Hendra virus (HeV) and Nipah virus (NiV) belong to the genus *Henipavirus* in the family *Paramyxoviridae*. Henipavirus infections were first reported in the 1990’s causing severe and often fatal outbreaks in domestic animals and humans in Southeast Asia and Australia. NiV infections were observed in humans in Bangladesh, India and in the first outbreak in Malaysia, where pigs were also infected. HeV infections occurred in horses in the North-Eastern regions of Australia, with singular transmission events to humans. Bats of the genus *Pteropus* have been identified as the reservoir hosts for henipaviruses. Molecular and serological indications for the presence of henipa-like viruses in African fruit bats, pigs and humans have been published recently. In our study, truncated forms of HeV and NiV attachment (G) proteins as well as the full-length NiV nucleocapsid (N) protein were expressed using different expression systems. Based on these recombinant proteins, Enzyme-linked Immunosorbent Assays (ELISA) were developed for the detection of HeV or NiV specific antibodies in porcine serum samples. We used the NiV N ELISA for initial serum screening considering the general reactivity against henipaviruses. The G protein based ELISAs enabled the differentiation between HeV and NiV infections, since as expected, the sera displayed higher reactivity with the respective homologous antigens. In the future, these assays will present valuable tools for serosurveillance of swine and possibly other livestock or wildlife species in affected areas. Such studies will help assessing the potential risk for human and animal health worldwide by elucidating the distribution of henipaviruses.

## Introduction

Hendra virus (HeV) and Nipah virus (NiV) represent the prototypes of the genus *Henipavirus* within the family *Paramyxoviridae*. Henipaviruses first emerged in Southeast Asia and Australia in the 1990’s, causing severe febrile illness in domestic animals and humans [1–3]. Flying foxes of the genus *Pteropus* have been identified as the major natural reservoir of these zoonotic viruses [4, 5]. Virus transmission mainly occurred from bats to intermediate hosts such as pigs or horses, before humans were eventually infected by contact to these intermediate hosts [6–9]. However, during more recent NiV outbreaks in Bangladesh and India, direct transmission from bats to humans and human-to-human transmission also occurred [10, 11]. Both viruses require handling under Biosafety Level 4 (BSL 4) conditions. The diagnostics of acute HeV or NiV infections primarily relies on a direct detection of the viral agent via molecular assays such as real-time RT-PCR, immunohistochemistry or virus isolation [12]. However, since a broad variety of mammalian species have been shown to be susceptible to HeV or NiV infection under experimental conditions, serosurveillance studies in affected areas may play an important role in improving our understanding of the epidemiology of these infections [13–20]. For these studies, simple and cost-efficient serological diagnostic assays are needed that can easily be performed outside a BSL 4 facility. In the past, several strategies have been followed to express recombinant henipavirus proteins that can be handled under BSL 2 conditions either in indirect enzyme-linked immunosorbent assay (ELISA) or in Luminex-based multiplexed microsphere assays [21–27]. Data in several reports indicated that there are cross-reactive antibodies in serum samples from domestic animals and livestock not only in the Southeast Asian / Australian region, but also in geographic areas where henipavirus infections have not been reported, such as Sub-Saharan Africa [28–32]. In areas of Bangladesh where human NiV outbreaks had been observed, serum samples from pigs, cattle and goats have been tested positive for the presence of antibodies against a truncated, soluble form of the NiV glycoprotein (NiV sG) in a Luminex-based microsphere assay [31].

In this study, glycoproteins of HeV and NiV (sHeV G; sNiV G), as well as the NiV nucleocapsid protein (NiV N) were produced for the development of indirect ELISAs. Both viral proteins were selected due to their known immunogenicity. The G proteins were expressed in the eukaryotic parasite *Leishmania tarentolae* (*L*. *tarentolae*), whereas the NiV N protein was expressed in *Escherichia coli* (*E*. *coli*). Since HeV and NiV N proteins share a homology of 92% at the amino acid level [33], we used the NiV N ELISA for initial serum screening regarding the general reactivity of the tested sera with henipaviruses. Subsequently, the serum samples were tested on both G protein based ELISA assays to differentiate between HeV and NiV infections. To evaluate the antigens for their suitability in ELISA, we used a number of henipavirus IgG negative porcine sera and a panel of well characterized sera from experimentally HeV or NiV infected pigs.

## Material and methods

### Expression of his-tagged NiV nucleocapsid (N) protein

The NipahN/IRES/CMV plasmid was kindly provided by Dr. Markus Czub (Public Health Agency of Canada, Winnipeg, Manitoba, Canada), and the N-protein coding region was sub-cloned using the *Bam*H I restriction sites into the pET-30a vector (Novagen), creating the pET-30a/Nipah N plasmid. After confirmation of positive pET-30a/Nipah N clones by sequencing using the commercially available primers T7 FOR promoter- 5’-TAA TAC GAC TCA CTA TAG G_3’ and T7 Terminator Primer -5’-CCG CTG AGC AAT AAC TAG C-3’ 8 (Millipore Sigma) with subsequent comparison to the NiV genome (accession no. AF212302), the plasmid was transformed into the Rosetta (DE3) *E*. *coli* bacterial cell line (Novagen) for expression. The inclusion body fraction contained most of the N protein. The harvested inclusion bodies were solubilized using standard methods, and the inclusion body proteins were separated by 10% sodium dodecyl sulfate-polyacrylamide gel electrophoresis (SDS-PAGE) on large 20 cm PROTEAN II xi (Bio-Rad) apparatus. Each gel was washed in Milli-Q water and stained with a solution of 0.2 M copper (II) chloride hydrate for 10 min. The largest band (~62 kDa) was cut-out and destained in Towbin’s buffer [34]. Destained gel was electro-eluted using the Bio-Rad Model 422 Electro-Eluter according to the manufacturer’s instructions. The membrane filter used had a molecular weight cut-off of 15 kDa. After electro-elution for 4 h at a constant current of 10 mA per holder, samples were aliquoted in 100 μl fractions and stored at -20°C.

### Immunoblot analysis of NiV N antigen

The NiV N protein was characterized by immunoblot using standard procedures. Briefly, N antigen was separated by 10% SDS-PAGE. After transfer to a polyvinylidene fluoride (PVDF) membrane, the membrane was blocked with 5% skim milk (Gibco, Germany) diluted in PBS/0.05%Tween-20 (PBST) for 1 h at room temperature (RT), and probed overnight at 4°C with NiV N protein specific monoclonal antibody F45G4 was diluted to 1:2,000 [35]. After washing with PBST, the membrane was incubated for 1 h at RT with an anti-mouse secondary antibody conjugated to horseradish peroxidase (HRP; Sigma-Aldrich) in at a 1:3,000 dilution. The blot was then incubated with SuperSignal West Pico Chemiluminescent Substrate according to the manufacturer’s guidelines (Thermo Scientific), and protein detection was visualized on Amersham Hyperfilm ECL ^™^ film (Fisher Scientific).

### Transmission electron microscopy of NiV N antigen

An inclusion body fraction of semi-purified Nipah N antigen was prepared by negative contrast electron microscopy. Specifically, 20 μl of sample was absorbed to formvar-coated carbon-stabilized copper grids and stained with 2% phosphotungstic acid, pH 7.2 (wt/vol). The specimen grids were examined in a Philips CM 120 transmission electron microscope, operating at an accelerating voltage of 80 kV. Micrographs were taken between 28,000X -45,000X using Kodak Electron Microscope Film 4489. The negatives were scanned using an Epson Perfection 3200 photo scanner and enlarged 2.5X.

### Expression of HeV and NiV attachment (G) proteins in *Leishmania tarentolae* (*L*. *tarentolae*)

The expression of a truncated soluble HeV G (sHeV G) protein has been described before [36], and the expression of a truncated soluble NiV G (sNiV G) protein in the *L*. *tarentolae* system (Jena Bioscience, Germany) was performed accordingly. Briefly, the NiV G sequence was codon optimized for the codon bias of *L*. *tarentolae* and the transmembrane domain and cytoplasmic tail were replaced by a double *Strep*-tag in order to enable affinity chromatography. The sequence product was cloned into the vector pLEXSY-sat2 (Jena Bioscience) using *Xba*I and *Not*I restriction sites to yield pLEXSYNiVG. The codon-optimized NiV G sequence was submitted to GenBank (accession No. MF379666). Transfection, clonal selection and subsequent protein purification were carried out as described previously [36]. Purity and size of the protein was evaluated by 10% SDS-PAGE and subsequent Coomassie blue staining. Protein concentration was determined by modified Bradford protein assay based on bovine serum albumin (BSA; GE Healthcare, Germany) as standard and according to the manufacturer’s instructions (Sigma Aldrich).

### Generation of monoclonal antibody (mAb) 5G1B1 raised against the sHeV G protein

Female BALB/c mice were immunized five times intraperitoneally with 15 μg of recombinant HeV G protein expressed in the *L*. *tarentolae* system mixed with an equal amount of GERBU Adjuvant MM (GERBU Biotechnik GmbH) in an interval of 4 weeks. Four days after the final boost, the immunized mice were euthanized and the spleens were removed under aseptic conditions. Splenocytes were harvested in serum-free RPMI-1640 medium (Invitrogen/Thermo Scientific) by using a cell strainer (BD Biosciences). Murine myeloma SP2/0 cells (Cell Culture Collection of the Friedrich-Loeffler-Institut, Germany) and the isolated splenocytes were fused in presence of polyethylene glycol 1500 (Roche Applied Science) following a standard protocol [37, 38] by using a cell-to-cell ratio of 4:1. Afterwards, 10.5x10^6^ fused spleen cells were seeded in three different cell densities (30,000, 15,000, and 7,500 spleen cells per well, two plates per density) in 96 well flat-bottom plates (Greiner bio-one) and incubated over 10 days at 37 °C, 90% RH, and 5% CO_2_. The complete RPMI-1640 culture medium contained 10% fetal calf serum (Fischer Scientific, #11573397), 1x MEM non-essential amino acids, 2 mM L-glutamine, and 1 mM sodium pyruvate (Invitrogen/Thermo Scientific). For the initial growing phase of the hybridoma cultures, the complete medium was additionally supplemented with 1x BM Condimed H1 (Hybridoma Cloning Supplement, Sigma-Aldrich) as well as 1x HAT Media Supplement (50×) Hybri-Max (Sigma-Aldrich) for selection. Growing cultures were screened for specific antibodies by ELISA using the recombinant protein. Cells from positive cultures were cloned at least twice by limiting dilution (0.1 cells per well) for generating monoclonal antibody producing cell clones [complete RPMI-1640 medium supplemented with 1x HAT Media Supplement (50×) Hybri-Max (Sigma-Aldrich)]. Final clones were adapted to complete RPMI-1640 medium without any further supplements.

### Immunofluorescence assay (IFA) for the detection of HeV and NiV G antigens

For immunofluorescence assay (IFA), Vero76 cells (Cell Culture Collection of the Friedrich-Loeffler-Institut, Germany) were either transfected with the pCAGGS HeV G plasmid [36] or with a codon-optimized NiV G expression plasmid (pCAGGS NiV G; GeneArt) using *Trans*IT-LT1 transfection reagent (Mirus Bio). After 48 h, monolayer cultures were fixed with 4% paraformaldehyde solution and permeabilized with 0.2% Triton-X100 (Sigma–Aldrich) in PBS. For the detection of both proteins, undiluted hybridoma supernatant producing the mAb 5G1B1 was used followed by the incubation with Alexa Flour 488-conjugated secondary anti-mouse antibodies (1:500 in 5% BSA in PBS; 5% BSA/PBS). Fluorescence was visualized using a DMI7 live cell microscope (Leica), a CSU-W1-T spinning disc confocal scanning head (Yokogawa) and an iXon Ultra 888 EMCCD camera (Andor).

### Indirect ELISA based on NiV N protein

Electro-eluted NiV N antigen in 0.01 M PBS, pH 7.2 was coated on Nunc F flat bottom polystyrene plates at a concentration of 100 ng/well (100 μl volume). The plates were incubated overnight at 4°C followed by washing five times using PBST solution. Each well was then blocked using a volume of 100 μl/well of blocking buffer (3% BSA/10% horse serum/0.1% Tween 20 in 0.01 M PBS pH 7.2) buffer. Sterile Donor Horse Serum (lot#CS-C14-500) was purchased in 2002 from CanSera. After incubating the plates at 37°C for a minimum of 1 h with shaking, the plates were washed as outlined above. Titration experiments revealed a 1:100 dilution of pig serum (both negative and test sera) in blocking buffer as optimal for this ELISA system. (Negative serum samples were submitted to NCFAD for pseudorabies and classical swine fever testing (NCFAD collection of diagnostic samples); test serum samples were collected during the animal studies mentioned in the Ethics statement. After adding 100 μl/well, the plates were incubated at 37°C for 1 h with shaking, followed by a washing step as outlined previously. A 1:1,000 dilution (in blocking buffer) of rabbit anti-pig IgG secondary antibody conjugated to HRP (100 μl/well) (Intermedico) was then added to the plates for 1 h at 37°C with shaking. After another washing step, 100 μl/well of enzyme substrate (2,2’-azino-bis(3-ethylbenzothiazoline-6-sulphonic acid)) (ABTS; Roche Diagnostics) was added. After incubating the plates for 15 min at RT in the dark, they were analyzed on a microtiter reader at an absorbance of 405 nm.

### Indirect ELISA based on sHeV and sNiV G proteins

sHeV and sNiV G proteins were diluted in 0.01 M PBS, pH 7.4 and coated on Medisorp 96 well plates at a concentration of 100 ng/well (100 μl volume) and kept at 4°C overnight. Extracts from untransfected *L*. *tarentolae* served as mock antigens in control wells to evaluate unspecific binding of the sera. Plates were blocked with 5% skim milk in 0.01 M PBS for 2 h at 37°C and washed three times with PBST. Sera were diluted 1:200 in 2.5% skim milk in PBST and added in duplicate to the control and antigen containing wells. After an incubation at 37°C for 1 h, the plate was washed again three times. Goat-anti-swine IgG HRP conjugate (Dianova) was added in a dilution of 1:10,000 and incubated for 1 h at 37°C. After four washes with PBST, 3,3’,5,5’-Tetramethylbenzidine (TMB) peroxidase substrate (Bio-Rad, Munich) was added to the wells for color development and stopped after 10 min at RT with equal amounts of 1 M sulfuric acid. Absorbance was measured at 450 against 590 nm in a Tecan Infinite 200Pro ELISA Reader (Tecan Deutschland GmbH).

### Serum samples and statistical analysis

For the evaluation of the NiV N protein based ELISA, 239 serum samples submitted to NCFAD for pseudorabies and classical swine fever testing (NCFAD collection of diagnostic samples) were used as a negative control panel. For the evaluation of the ELISA assays based on the sHeV and sNiV G proteins, serum samples from 154 pigs from different holdings in Germany (collected during the animal studies mentioned in the Ethics statement) were used as the negative control panel since for both Canada and Germany there is no history of previous henipavirus infection. A panel of 12 sera from pigs experimentally infected with NiV (10 sera) or HeV (2 sera) served as a positive control panel for the initial evaluation of all three ELISAs. These positive sera were collected during experimental NiV and HeV infection trials from different days post infection (dpi), therefore displaying different titers in confirmatory plaque reduction neutralization test (PRNT). Sera were heat inactivated at 56°C for 60 min prior to analysis in the ELISA. The mean values, standard deviation and diagnostic specificity (D-SP) values were calculated based on the absorbance values of the serum samples. The D-SP was calculated using the following formula: number of negative sera tested minus number of negative sera tested positive divided by number of negative sera tested x 100. When determining the positivity of the pig serum samples tested, we used a cut-off absorbance of three standard deviations above the mean.

### Immunoblot analysis of ELISA-positive porcine serum samples

To confirm the specific binding of the ELISA-positive porcine serum samples to the antigens, an exemplary panel of porcine sera was investigated for their reactivity in immunoblot, using a cell lysate of HEK-293T cells (Cell Culture Collection of the Friedrich-Loeffler-Institut, Germany) transfected with a codon optimized NiV G expression plasmid (pCAGGS NiV G) as antigen. Immunoblots were performed as described above for the detection of the N protein. Briefly, cell lysate was separated by SDS-PAGE and transferred to a nitrocellulose membrane, which was then incubated with porcine sera (dilution 1:200 in 2.5% skim milk in PBST) overnight at 4°C. Species-specific goat anti-swine antibodies conjugated with HRP (Dianova) were incubated on the membrane for 1 h at RT in a 1:5,000 dilution. MAb 5G1B1 in a dilution of 1:100 served as a positive control for the detection of NiV G antigen expressed in HEK-293T cells.

### Plaque reduction neutralization test (PRNT)

The PRNT was performed as published previously using NiV [39], and all procedures with live virus were performed under BSL-4 conditions.

### Ethics statement

Manipulations of animals at the Friedrich-Loeffler-Institut mentioned in this report had the specific approval (LALLF 7221.3–2.5-004/10, LALLF M-V/TSD/7221.3–2.1.014/10, LALLF M-V/TSD/7221.3–2.1-017/13. and LALLF M-V/TSD/7221.3–1.1-022/13) from the competent authority of the Federal State of Mecklenburg-Western Pomerania, Germany, on the basis of national (Tierschutzgesetz, Tierschutz-Versuchstier-Verordnung) and European (RL 2010/63/EU) legislation, which also includes the Ethic Committee of Mecklenburg-Western Pomerania. In addition, animal studies are continuously monitored by the Animal Welfare Officer and were approved by the Institutional Animal Care and Use Committee (IACUC).

Animal housing and manipulations performed at the Canadian Food Inspection Agency met the Canadian Council on Animal Care guidelines and were approved by the Animal Care Committee of the Canadian Science Centre for Human and Animal Health under Animal Use Documents: #C-02-006, #C-04-005 and #C-08-008.

## Results

### Antigen production

**Fig 1:**
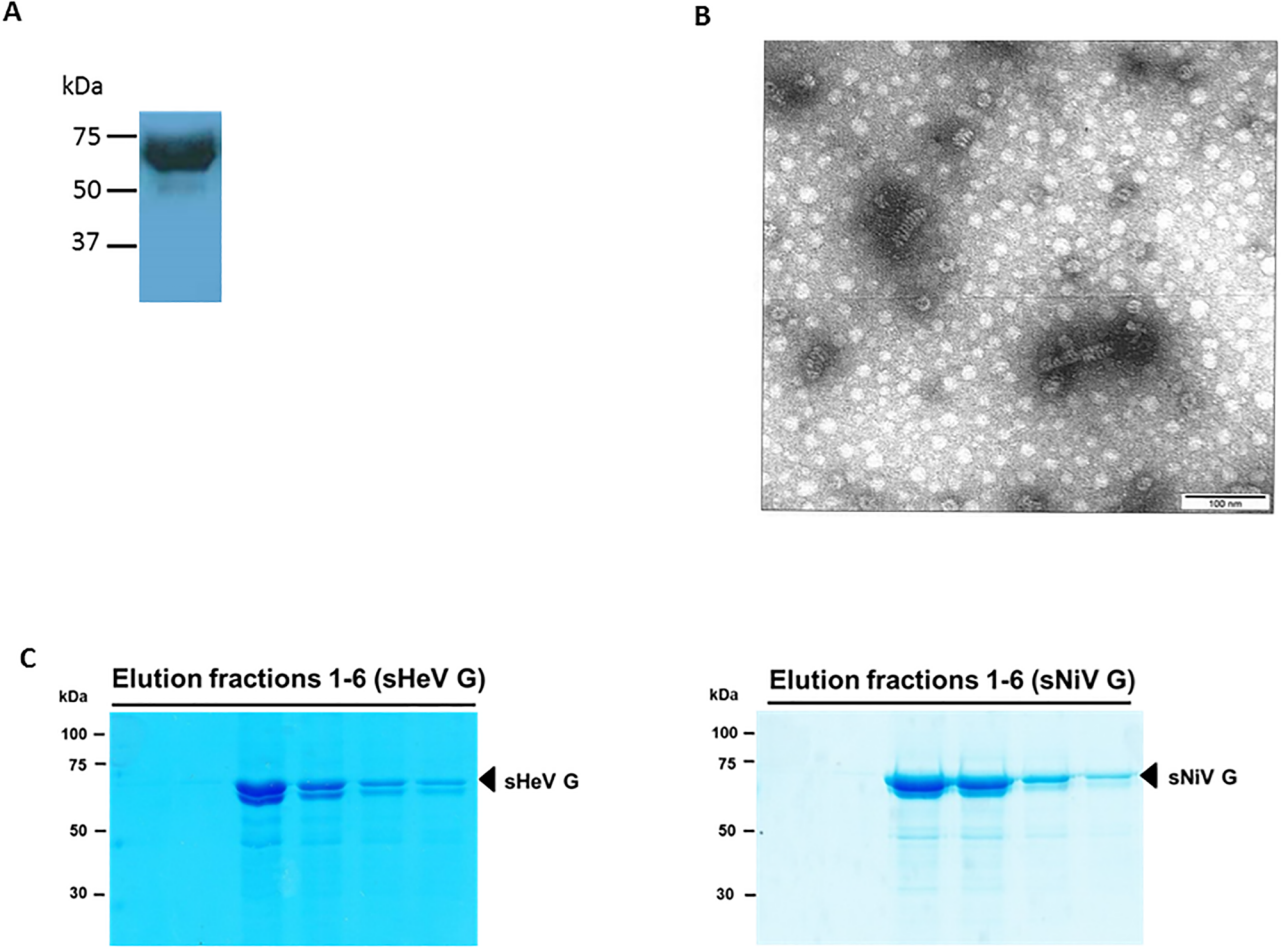
Purification and detection of ELISA antigens. **A**. Immunoblot of purified his-tagged NiV N expressed in *Escherichia coli*. The identity of the NiV N protein was confirmed by anti-N monoclonal antibody F45G4 using enhanced chemiluminescence. **B**. Negative contrast electron microscopy of an inclusion body fraction of semi-purified NiV N antigen. The specimen grids were examined in a Philips CM 120 transmission electron microscope, operating at an accelerating voltage of 80 kV. Micrographs were taken between 28,000X–45,000X using Kodak Electron Microscope Film 4489. The negatives were scanned using an Epson Perfection 3200 photo scanner and enlarged 2.5X. **C**. Coomassie staining of purified recombinant sHeV G and sNiV G protein elution fractions. Purification of Strep-tagged sHeV G (A) and sNiV G (B) from *Leishmania tarentolae* cell lysates was performed via Strep-tag affinity chromatography. Elution fractions were separated by 10% SDS-PAGE under reducing conditions and visualized by Coomassie staining.

Most of the NiV N protein was found in the insoluble fraction harvested from the of *E*.*coli* expression system. The protein was harvested by electro-elution from this fraction, and purified by gel electrophoreses. The identity of the harvested protein as NiV N was confirmed by immunoblotting with monoclonal anti-N antibody F45G4. The apparent molecular size of the recombinant NiV N protein corresponded with the expected molecular size of approximately 63 kDa (a 5 kDa histidine tag from the pET-30a vector included) (Fig 1A). As determined by Bradford analysis, a recovery of 6.72 mg of Nipah N protein was obtained per liter of a starting culture of Nipah N/pET30A transformed Rosetta *E*. *coli*. Transmission electron microscopy of a solubilized inclusion body pellet fraction (isolated after 2 h of induction) containing the NiV N protein, detected both clusters and individual herringbone-like structures (Fig 1B). These structures ranged in size from ~70–100 nm in length. Upon treatment of these structures with the detergent SDS (i.e. after electro-elution), no herringbone-like structures were apparent. A solubilized inclusion body pellet of the negative controls (isolated after 0 h of induction) did not show any such structures (S1 Fig).

To be able to compare the performances of sNiV G and sHeV G in ELISA, a Leishmania-based expression system was chosen in order to maintain proper glycosylation of the glycoproteins. After transfection of sequence-verified plasmids, four recombinant clones were randomly selected and used for sNiV G protein expression. As previously observed for the expression of the closely related sHeV G protein in *L*. *tarentolae*, sNiV G was not efficiently secreted into the growth medium, even though the protein was fused to the secretory signal peptide (SP) of *Leishmania mexicana* secreted acidic phosphatase (LMSAP). However, we purified nearly 1 mg of recombinant protein per liter of densely grown cell culture lysates as quantified by Bradford protein assay. The electrophoretic analysis of the six elution fractions of the two glycoproteins demonstrated the enrichment and purity of the approximately 70 kDa comprising sHeV G and sNiV G as depicted in Fig 1(C).

### Generation and characterization of monoclonal antibodies (mAb) raised against sHeV G

**Fig 2:**
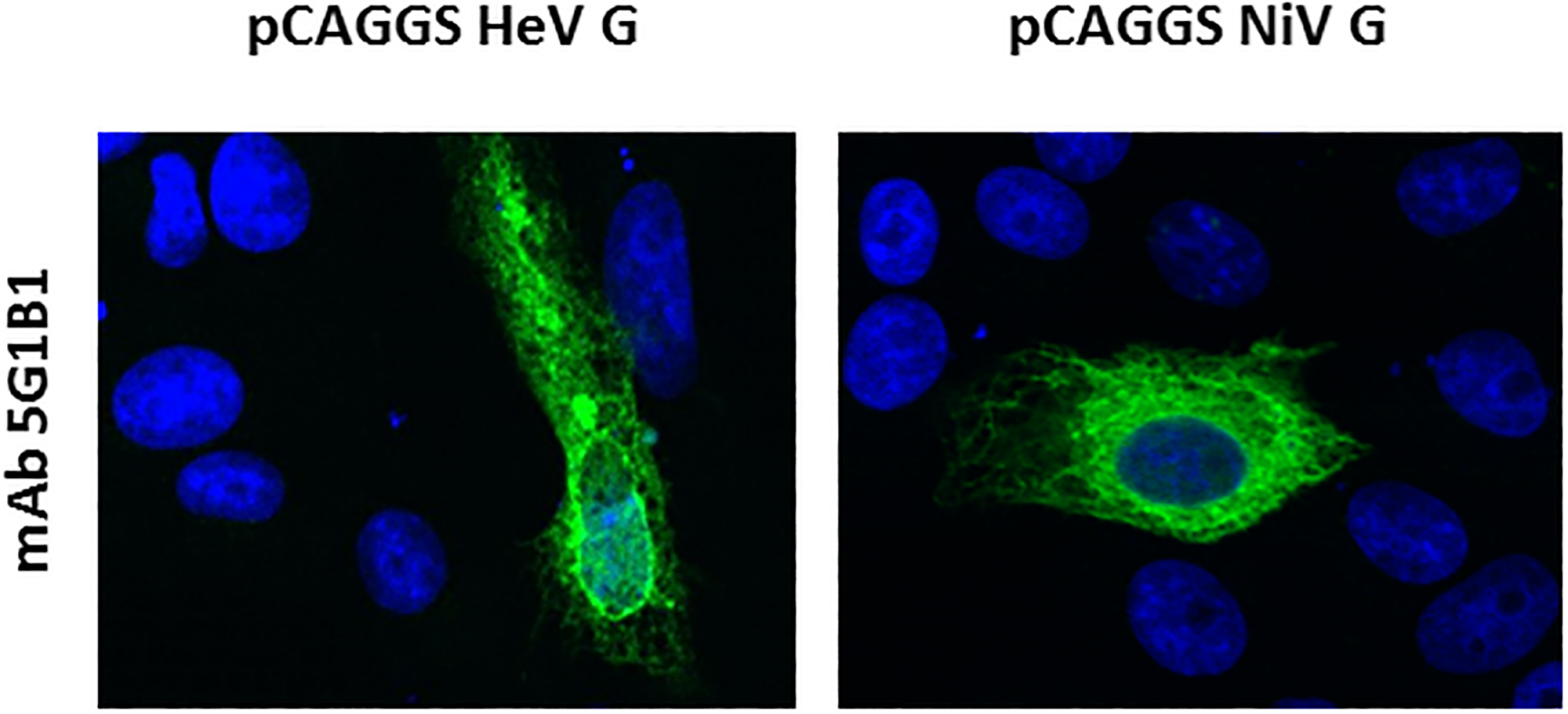
Indirect immunofluorescence analysis of cells transfected with HeV G and NiV G using mAb 5G1B1. Vero 76 cells were transfected with the indicated plasmids to express NiV and HeV G proteins. For immunostaining, the newly generated cross-reactive monoclonal antibody 5G1B1 was used as well as the respective polyclonal mice serum as positive control followed by mouse specific Alexa-Fluor 488-labeled secondary antibodies. Nuclei were stained with Hoechst. Fluorescence was visualized by a DMI7 live cell microscope (Leica), magnification 630 x.

For the generation of monoclonal antibody producing hybridoma clones, BALB/c mice were immunized with the Leishmania-derived sHeV G protein. Growing cultures were screened for specific antibodies by Western Blot using the recombinant sHeV G protein and a commercially available recombinant form of the HeV G protein expressed in mammalian cells (Sino Biological Inc., USA; Panel A in S2 File). One most promising mAb clone designated 5G1B1 was selected eventually and further validated regarding the specific binding by IFA in Vero 76 cells that were either transfected with plasmid-derived HeV G or NiV G. MAb 5G1B1 showed a highly specific detection of the HeV and NiV G proteins in transfected Vero 76 cells (Fig 2). These results indicate a high level of conservation between the targeted epitope of both G proteins. By that we were able to show that 48 h after transfection, NiV and HeV G proteins both accumulated on the cell surface as well as in the cytoplasm. In contrast, no signal was observed in mock transfected cells (Panel B in S2 File).

The reactivity of clone 5G1B1 was also confirmed by Western blot analysis of NiV G protein expressed in HEK-293T cells as well as sHeV G and sNiV G expressed in *L*. *tarentolae* (Panel C in S2 File).

### Application of recombinant proteins in ELISA

**Fig 3:**
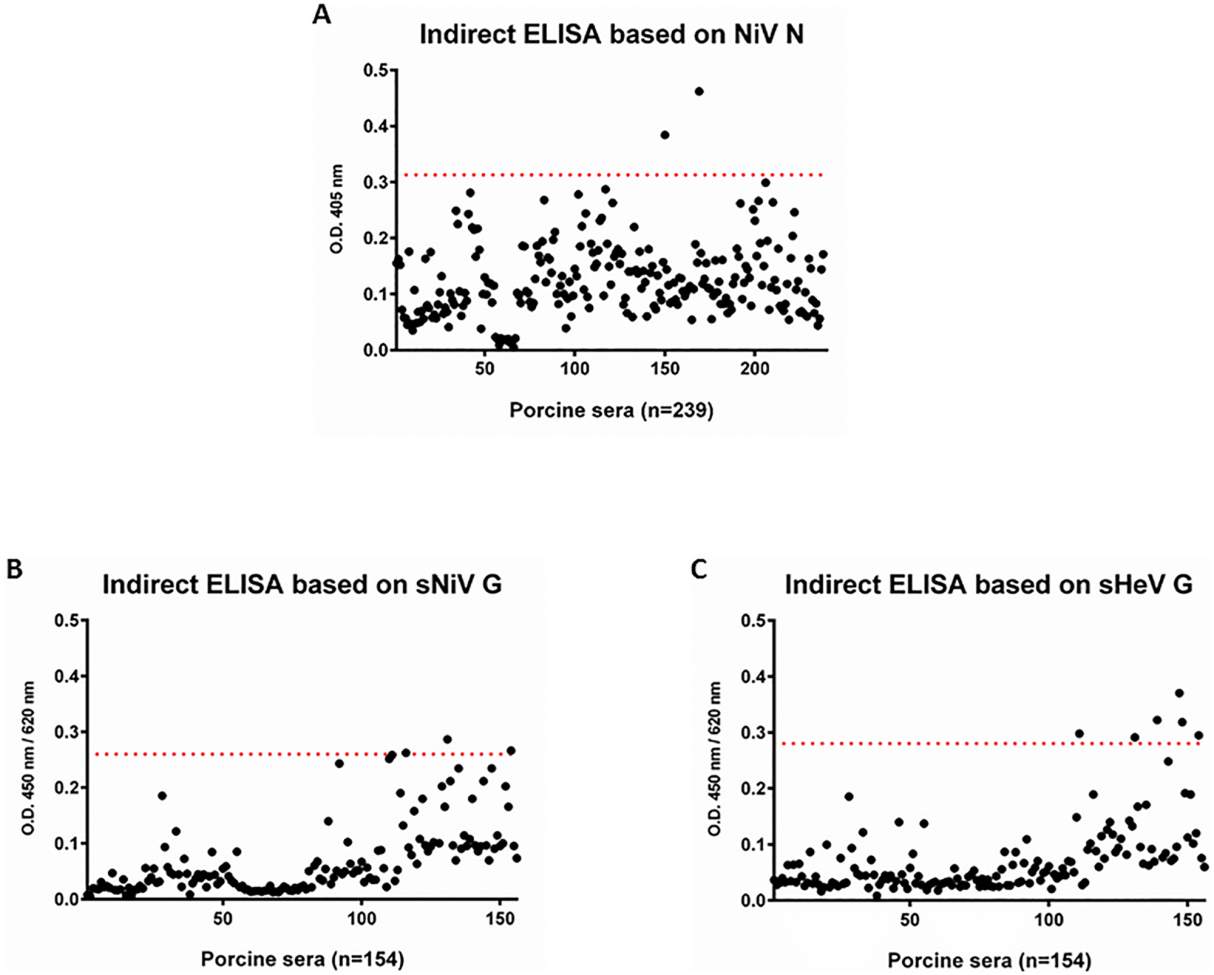
Cut-off determination for the three independent ELISAs. Cut-off values were evaluated by testing of 239 negative control sera (NiV N based ELISA) and 154 sera (sHeV and sNiV G based ELISAs). The horizontal red broken line represents the cut-off value defined as mean OD value of all tested negative control sera plus three standard deviations. OD values were measured at 450 nm for the sHeV G and sNiV G as well as at 405 nm for the NiV N based ELISAs.

In order to determine diagnostic specificity of the NiV N ELISA, 239 negative porcine sera submitted to NCFAD for pseudorabies and classical swine fever testing were used as negative control panel. These sera were considered negative due to the lack of evidence for circulation of henipaviruses in Canada. With the cut-off value of the determined optical density at 405 nm (OD_405_) of 0.32 (average OD_405_ value was at 0.124, plus 3 StDev) the diagnostic specificity (D-SP) of the N ELISA was at 99% (Fig 3A). Only two serum samples had OD_405_ values above the cut-off, and would undergo confirmatory testing such as Western blot analysis or PRNT to determine whether they are true or false positive results.

**Fig 4:**
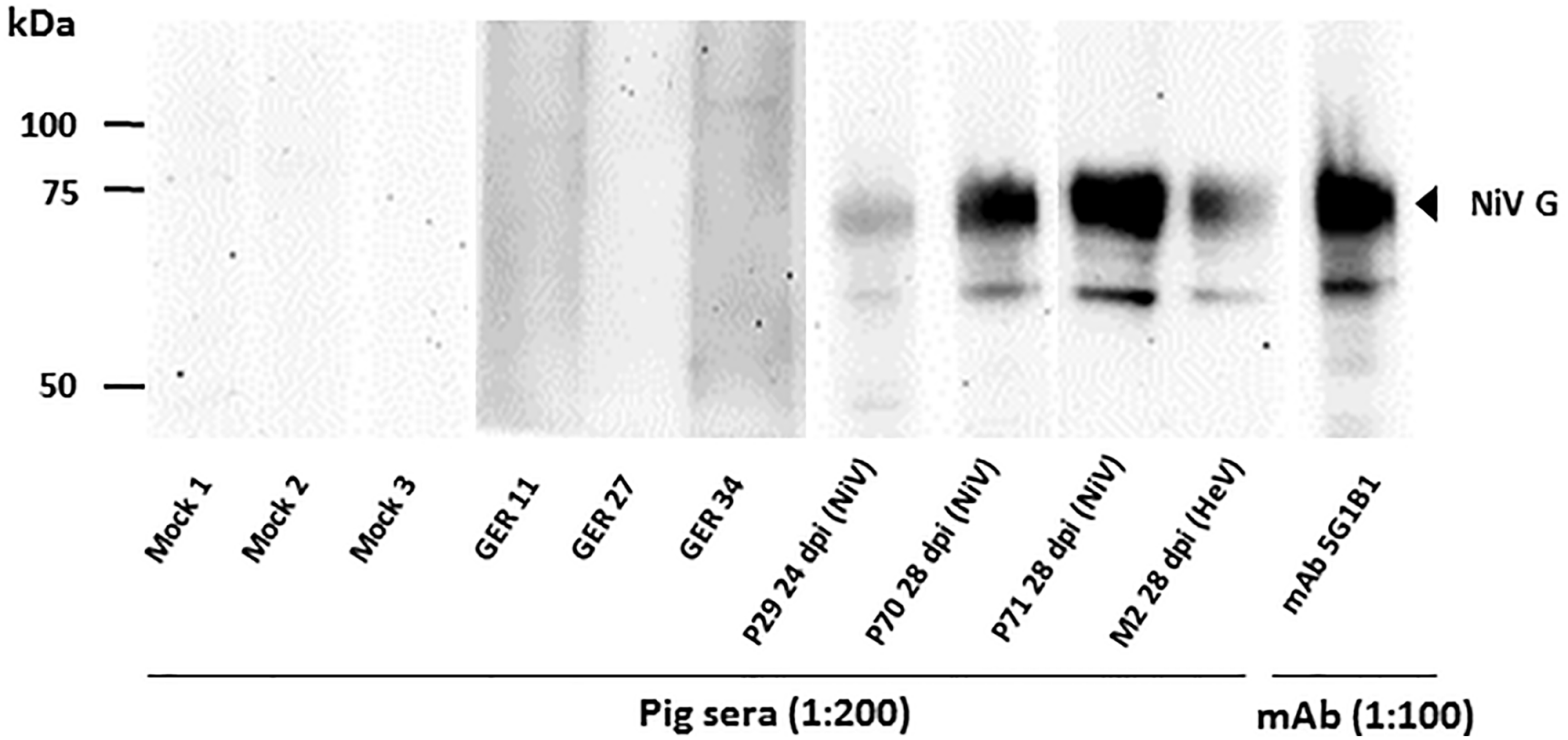
Immunoblot analysis of serum reactivity against plasmid derived NiV G. Serum samples from HeV or NiV infected pigs were collected at different dpi and tested for reactivity against the homologous and heterologous recombinant protein, respectively. Porcine sera from German pigs without history of henipavirus infection served as negative control (Mock 1–3). Two German pig sera (GER11 and GER34) that exceeded the calculated cut-off value in both G based ELISAs as well as one serum (GER27) that only exceeded the cut-off value in the sHeV G based ELISA were tested exemplarily for reactivity in immunoblot analysis. All sera were diluted as indicated. The monoclonal antibody 5G1B1 was utilized in a dilution of 1:100.

For the evaluation of both sHeV G and sNiV G ELISAs, we investigated 154 sera from pigs originating from different holdings in Germany. These sera were considered negative due to the lack of evidence for the circulation of henipaviruses in Germany. The OD_450_ average of all tested German pig sera in the sHeV G and sNiV G ELISA was 0.076 and 0.068, respectively, highlighting the generally low background of these tests. Using the average OD_450_ plus 3 StDev, cut-off values were calculated as 0.28 for the sHeV G and of 0.26 for the sNiV G ELISA (Fig 3B and 3C). The results obtained for six sera in the sHeV G ELISA and three sera in the sNiV G ELISA slightly exceeded the calculated cut-off values. Six of these samples were tested by Western Blot against a cell lysate of transfected HEK-293T cells expressing the NiV G protein with negative result (Fig 4 and S3 Fig) and were thus considered as false positive. Consequently, a D-SP of 96.1% and 98.0% was determined. Although due to lack of sufficiently characterized samples, this immunoblot assays could not be fully validated, the positive control monoclonal antibody 5G1B1 raised against the sHeV G protein clearly cross-reacted with the NiV G protein expressed in HEK-293T cells in immunoblot test. Sera obtained from HeV or NiV challenge studies also clearly (cross-) reacted with the NiV G protein in the immunoblot (Fig 4). In contrast, two German pig sera (GER11 and GER34) that exceeded the calculated cut-off value in both G based ELISAs, as well as one serum (GER27) that only exceeded the cut-off value in the sHeV G based ELISA did not display any reaction with the antigen in immunoblot analysis.

**Fig 5:**
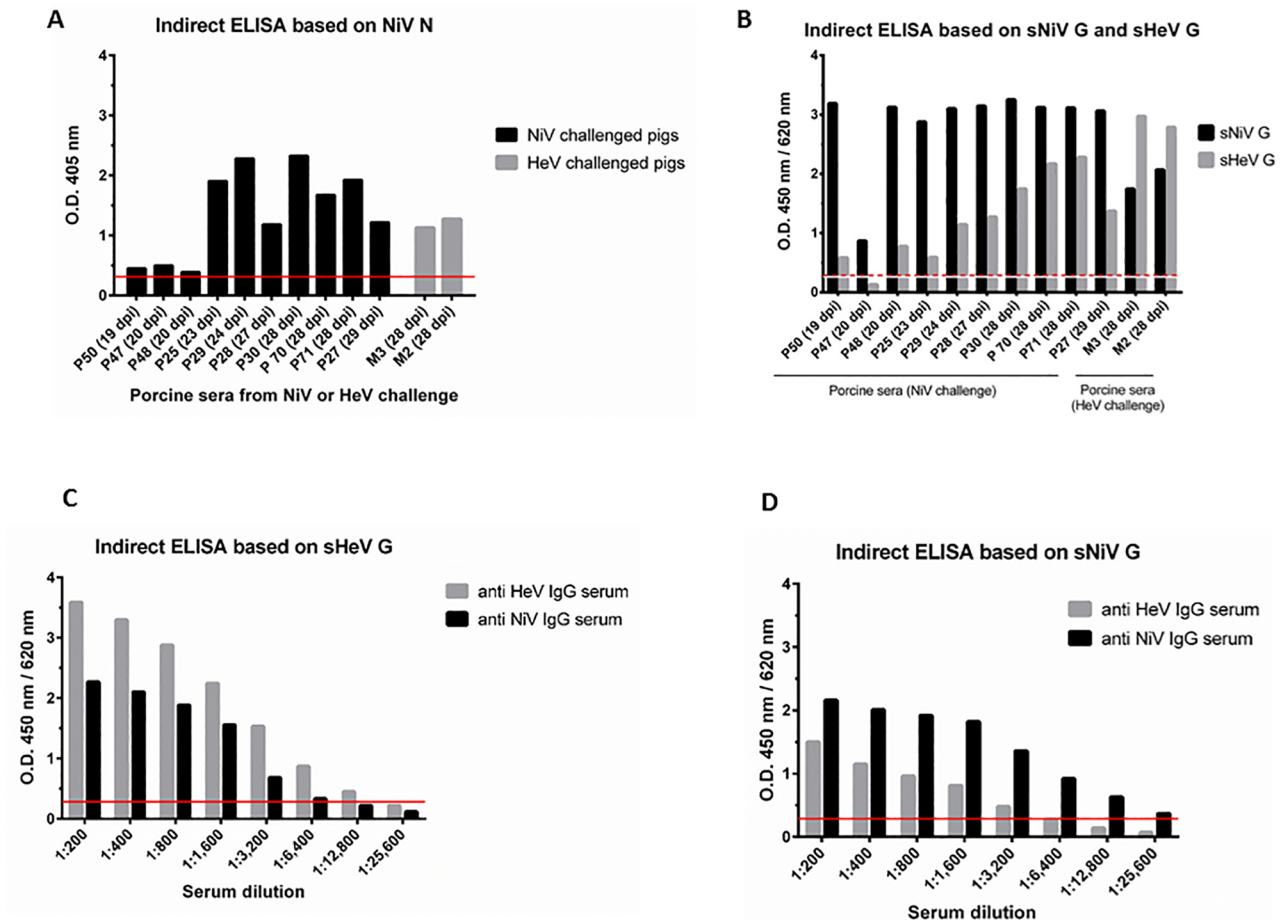
Serum reactivity of experimentally henipavirus infected pigs in ELISAs based on soluble HeV and NiV glycoproteins and NiV nucleoprotein (sHeV/sNiV G; NiV N). **A**. Sera from pigs (P) and minipigs (M) were collected at different days post infection (dpi) and tested for the presence of henipavirus specific IgG in the N based assay, with the red line representing the cut-off value. **B**. Serum reactivity was confirmed and specified in the sHeV G and sNiV G specific ELISAs with the white line representing the cut-off value of the sNiV G based assay and the red broken line representing the sHeV G assay cut-off value. **C, D**. One HeV IgG positive as well as one NiV IgG positive serum were serially titrated and tested for reactivity in the sHeV G based assay (C) and in the NiV based assay (D) with the red lines representing the cut-off values.

To test the general suitability of our assay system for the detection of henipavirus-infected pigs, we analyzed a set of serum samples that had been collected during NiV and HeV challenge studies and which were determined positive for neutralizing antibodies by PRNT [14, 39–41]. These serum samples were first screened using the NiV N based ELISA (Fig 5A), and then tested for their specific reactivity against the sHeV and sNiV G proteins (Fig 5B). As expected, the recombinant proteins showed stronger reactions with sera from pigs that were infected with the homologous virus: OD_450_ values of sera from NiV infected pigs were higher on the sNiV G ELISA as compared to the sHeV G ELISA, and vice versa. All the tested sera however clearly cross-reacted with the sHeV G or the sNiV G antigen, as illustrated in Fig 5B. Selected serum samples were also tested by NiV-PRNT to analyze the correlation between the results obtained by NiV N ELISA, NiV and HeV G ELISAs and NiV-PRNT, which showed an overall good correlation between the G protein based ELISAs and the PRNT (Table 1). For the sHeV G ELISA, one HeV IgG positive serum as well as one NiV IgG positive serum, both collected 28 dpi, were serially diluted for a better comparison of the binding affinities of the sera to the homologous and heterologous G proteins, respectively. For the serum of the HeV challenged pig (NiV-PRNT titer of 1,280), a serum dilution of 1:12,800 still revealed a positive result in the sHeV G ELISA (Fig 5C). The same PRNT titer (1,280) serum from a NiV infected pig displayed a negative result in the sHeV G ELISA in a 1:12,800 dilution, and a very weakly positive OD_450_ value at a dilution of 1:6,400 (Fig 5C).

**Table 1:** NiV N, NiV G and HeV G ELISA OD values and NiV-PRNT titers for selected serum samples collected from pigs experimentally challenged with NiV or HeV. These results confirm the correlation between the PRNT titers and the OD values determined in the G protein based ELISAs, as neutralizing antibodies are mostly directed against the viral glycoproteins. n.d. = not done.

sample ID (days post infection)	OD NiV N ELISA	OD sNiV G ELISA	OD sHeV G ELISA	NiV-PRNT titer
**P50** (19 dpi NiV)	0.453	3.189	0.585	n.d.
**P47** (20 dpi NiV)	0.500	0.869	0.132	n.d.
**P48** (20 dpi NiV)	0.389	3.125	0.777	n.d.
**P25** (23 dpi NiV)	1.906	2.877	0.592	1:20
**P29** (24 dpi NiV)	2.282	3.102	1.145	1:640
**P28** (27 dpi NiV)	1.185	3.146	1.274	1:1,280
**P30** (28 dpi NiV)	2.327	3.254	1.747	1:1,280
**P70** (28 dpi NiV)	1.676	3.122	2.169	> 1:160
**P71** (28 dpi NiV)	1.927	3.115	2.280	> 1:160
**P27** (29 dpi NiV)	1.219	3.061	1.372	1:640
**M3** (28 dpi HeV)	1.131	1.744	2.971	> 1:160
**M2** (28 dpi HeV)	1.275	2.068	2.787	> 1:160

## Discussion

Henipaviruses first emerged in the 1990’s leading to fatalities in Southeast Asian and Australian livestock and human population. HeV and NiV are highly pathogenic zoonotic viruses of bat origin and have caused several outbreaks with considerable economic and social impact [1, 42–45]. Serological surveillance to monitor the natural host and other susceptible animals represents a valuable approach to predict potential health risks. We developed a set of indirect ELISAs for swine samples to facilitate risk assessment in the affected areas. Recombinant HeV and NiV G and NiV N proteins were designed and evaluated for use in the ELISAs, as they do not, unlike whole virus antigens, require BSL 4 containment for production. We used a combination of these three recombinant proteins for an optimized detection and specification of antibodies directed against NiV, HeV or other paramyxoviruses in pigs. Although a recombinant NiV N protein expressed in *E*. *coli* has been used in serological assays earlier [23, 25], the combination of an *E*. *coli* derived NiV N protein and both HeV and NiV glycoproteins expressed in *L*. *tarentolae* to ensure a mammalian-like glycosylation pattern, helps us assessing and specifying the detected reactivity. While we use the recombinant NiV N protein for an initial screening of pig sera, reactive sera will be further examined using both recombinant glycoproteins. Due to the higher binding affinity to the homologous protein, this will ensure the differentiation between antibodies directed against NiV, HeV or other related paramyxoviruses.

Besides the safe production under BSL 2 conditions, recombinant proteins can be produced with a high reproducibility among antigen batches and facilitate standardization of assays [25]. To date, most of the recombinant proteins used in diagnostic assays are expressed in *E*. *coli*, baculovirus infected insect cells, or mammalian cells. In this study we used the highly efficient *E*. *coli* expression system for the NiV N protein. New expression systems have recently been developed especially for the expression of glycoproteins with mammalian-like glycosylation pattern, such as the eukaryotic parasite *L*. *tarentolae* that may serve as a valuable platform for viral protein expression due to their relatively easy maintenance and fast growth as well as high protein yields [46]. Therefore, in this study we used the highly efficient *L*. *tarentolae* expression system for the NiV G and HeV G proteins. Due to their immunogenicity, Leishmania-derived hepatitis C virus envelope glycoprotein and influenza virus hemagglutinin have been proposed as promising vaccine candidates [47, 48], and a truncated hepatitis E capsid protein was used in an indirect ELISA [49]. In our study, the sHeV G protein produced in *L*. *tarentolae* was not only successfully applied in a serological test but was also used to generate monoclonal antibodies. The selected clone 5G1B1 displayed a clear cross-reactivity against the NiV G protein in all conducted assays, including ELISA, IFA and immunoblots, indicating a target epitope in the G protein that is conserved between the two viruses.

In this report, the protein yield of sHeV G and sNiV G of approximately 500 μg– 1 mg per liter of densely grown Leishmania cells was within the expected range of the expression system [50]. In a previous study, NiV G was expressed in baculovirus-infected insect cells resulting in a less complexly glycosylated protein while obtaining higher protein yields of 1–2 mg/ml per 1.5 x 10^6^ Sf9 cells [21]. For the NiV N, a recovery of 6.72 mg of NiV N protein was obtained per liter of transformed Rosetta *E*. *coli* starting culture, which was considerably higher than what has been described in earlier studies using recombinant NiV N protein in *E*. *coli* obtaining yields of 1 mg per liter starting culture [25] and 0.8 mg per liter starting culture [51]. In another study, baculovirus-infected Sf9 cells were used for the expression of the recombinant NiV N protein at high yields of 2–3 mg per 1 x 10^6^ infected Sf9 cells [23]. Furthermore, when the NiV N protein was expressed in *E*.*coli*, the recombinant protein self-assembled into herringbone-like particles as observed before [52]. These results clearly indicate the bacterial expression system to be a quick and relatively easy method for the expression of high yields of recombinant NiV N protein in its native form.

The henipavirus G and N proteins were chosen due to their described immunogenicity [53, 25]. The henipavirus G protein is responsible for host cell receptor binding and thus a major target for neutralizing antibodies [54, 55]. HeV and NiV G proteins share 83% of amino acid sequence homology, whereas the N proteins of both viruses share up to 92% sequence homology, and serological cross-reactivity was confirmed by several studies, making the N protein based assay a good candidate for the initial screening of field sera [56, 33, 53, 35, 26, 14]. In a recent study by Marsh *et al*., rabbit antibodies against the N protein of the Cedar virus, another member of the *Henipavirus* genus, cross-reacted with the HeV N and NiV N in an immunofluorescence assay, although only a 58–59% amino acid sequence identity was observed [57]. These findings further highlight the potential of the N based ELISA as an initial henipavirus screening test. Another group investigated 76 bat sera from 11 different species from Brazil for reactivity in their newly established Nipah N based ELISA [58]. Interestingly, nine bat sera cross-reacted with the Nipah N protein in the ELISA as well as in the confirmatory IFA, indicating the circulation of henipa-like viruses in Brazil.

We calculated specificities of 99%, 96% and 98% for our ELISA based on NiV N, HeV G and NiV G. In recent studies using the NiV N protein, a specificity of 98.7% was calculated based on a sample panel of 1709 porcine positive and negative samples [59], while a specificity of 98.4% was determined by testing a set of 133 positive and negative human samples [25].

The observed broad cross-reactivity of antibodies against the N protein within the known members of the genus *Henipavirus*, however, necessitates additional serological assays specific for HeV or NiV, the viruses of human and animal health interest. Therefore, we developed a second ELISA based on either the sHeV or sNiV G proteins, in order to determine the specific reactivity of the serum.

We observed a considerable cross-reactivity of the sera against the heterologous antigens in ELISA which is in accordance with the cross-reactive NiV-PRNT results obtained from a HeV challenge study in pigs [14]. Interestingly, the cross-reactivity is not always correlated with cross-protection. For several animal models such as cats, African green monkeys and ferrets, animals that were pre-vaccinated with HeV G proteins were clearly cross-protected against a NiV challenge [60–63]. In pigs, however, a recent study revealed a lack of cross-protectivity and only low cross-neutralizing activity when vaccinated with one of the G proteins and challenged with the heterologous virus [64].

The ELISAs used in our study clearly discriminated sera from experimentally infected pigs collected at different dpi and sera from uninfected henipavirus negative pigs. However, since the number of available positive porcine field sera is very limited, the sensitivity of the assays can only be estimated. In addition, that applies equally to the confirmatory tests that were only validated by using serum samples from experimentally infected animals, while positive field sera were inaccessible for validation purposes. Therefore, the occurrence of false negative results when testing field sera cannot be completely ruled out for any of these tests. To finally validate the newly established assays, testing field sera of well-known origin would be desirable.

The recombinant proteins which were expressed in different expression systems obviously displayed the relevant antigenic epitopes necessary for the recognition in ELISA. A low number of assumed negative sera (i.e. originating from Canada or Germany, with no henipavirus activity) were reactive in individual ELISAs. These sera were further investigated by immunoblot or by NiV-PRNT [39], and no specific reaction was detected with the NiV G antigen expressed in eukaryotic HEK-293T cells or in the PRNT. Interestingly, there is recent serological and molecular evidence for the presence of a diverse group of henipa(-like) viruses in different geographic regions around the world [28, 29, 65, 66, 31, 67], even with reports about a potential spillover into the human population of Sub-Saharan Africa [68]. Although the collected data suggests a close antigenic relationship between the G proteins of HeV and NiV and that of the putatively circulating African henipavirus, it is noteworthy that these findings have not been linked to disease reports yet, and virus isolates are still missing. Chowdhury *et al*. tested a panel of serum samples from livestock located in a human NiV outbreak area with a considerable number of positive results in the applied Luminex assay using HeV and NiV G proteins as antigens [31]. Although none of the detected antibodies displayed neutralizing activities, the results emphasize the need for further surveillance, based on reliable serological diagnostics to better predict a potential risk to human health in the future. In the future, the newly established assays will be expanded and adapted to other mammalian species in different regions where henipa(-like) viruses have been assumed to occur. In our study, henipavirus G and N protein based ELISAs proved to be suitable assays for the detection of henipavirus specific antibodies in experimentally infected pigs. However, due to lack of availability of field sera and only a low number of available positive experimental sera, the ELISAs could not be fully validated in terms of diagnostic sensitivity. For their application in serological and epidemiological studies in pigs, it would be desirable to determine the assays’ detection limits in the early stage of infection. Nonetheless, these newly established tools will be highly valuable for a serological screening of different animal species in regions where natural reservoir hosts are abundant.

## Supporting information

S1 Fig Negative contrast electron microscopy of solubilized inclusion body pellet of the negative controls (isolated after 0 h of induction). The specimen grids were examined in a Philips CM 120 transmission electron microscope, operating at an accelerating voltage of 80 kV. Micrographs were taken between 28,000X–45,000X using Kodak Electron Microscope Film 4489. The negatives were scanned using an Epson Perfection 3200 photo scanner and enlarged 2.5X.
(TIF)

S2 Fig Reactivity of monoclonal antibody 5G1B1. **A**. Western blot analysis of 5G1B1 reactivity against commercially obtained HeV G/Fc (Sino Biologicals). The monoclonal antibody hybridoma supernatant 5G1B1 was utilized in a dilution of 1:10 and 1:100. Other hybridoma supernatants were tested but did not reveal positive signal in Western blot. **B**. Immunofluorescence analysis of monoclonal antibody 5G1B1 reactivity against Mock-transfected Vero76 cells. Vero 76 cells were transfected with the pCAGGS plasmid. For immunostaining, the newly generated cross-reactive monoclonal antibody 5G1B1 was used followed by mouse specific Alexa-Fluor 488-labeled secondary antibodies. Nuclei were stained with Hoechst. Fluorescence was visualized by a DMI7 live cell microscope (Leica), magnification 630 x. **C**. Western blot analysis of 5G1B1 reactivity against Leishmania-derived sHeV or NiV G. The monoclonal antibody hybridoma supernatant 5G1B1 was utilized in a dilution of 1:100.
(TIF)

S3 Fig Immunoblot analysis of reactivity of additional serum samples against plasmid derived NiV G. Serum sample from a NiV infected pig was collected at 7 dpi served as a positive control. Six German pig sera that exceeded the calculated cut-off values in or or both G based ELISAs were tested for reactivity in immunoblot analysis. All sera were diluted as indicated. The monoclonal antibody 5G1B1 was utilized in a dilution of 1:100.
(TIF)

## References

[1] Chua KB, Bellini WJ, Rota PA, Harcourt BH, Tamin A, Lam et al Nipah virus: a recently emergent deadly paramyxovirus. Science 2000; 288: 1432–1435. 10827955 10.1126/science.288.5470.1432

[2] Eaton BT, Broder CC, Middleton D, Wang LF. Hendra and Nipah viruses: different and dangerous. Nat Rev Microbiol. 2006; 4: 23–35. doi: 10.1038/nrmicro1323 16357858 10.1038/nrmicro1323 PMC7097447

[3] Han HJ, Wen HL, Zhou CM, Chen FF, Luo LM, Liu JW, et al Bats as reservoirs of severe emerging infectious diseases. Virus Res. 2015; 205: 1–6. doi: 10.1016/j.virusres.2015.05.006 25997928 10.1016/j.virusres.2015.05.006 PMC7132474

[4] Halpin K, Young PL, Field HE, Mackenzie JS. Isolation of Hendra virus from pteropid bats: a natural reservoir of Hendra virus. J Gen Virol. 2000; 81: 1927–1932. doi: 10.1099/0022-1317-81-8-1927 10900029 10.1099/0022-1317-81-8-1927

[5] Chua KB, Koh CL, Hooi PS, Wee KF, Khong JH, Chua BH, et al Isolation of Nipah virus from Malaysian Island flying-foxes. Microbes Infect. 2002; 4: 145–151. 11880045 10.1016/s1286-4579(01)01522-2

[6] Hanna JN, McBride WJ, Brookes DL, Shield J, Taylor CT, Smith IL, et al Hendra virus infection in a veterinarian. Med J Australia 2006; 185: 562–564. 17115969 10.5694/j.1326-5377.2006.tb00692.x PMC7168387

[7] Playford EG, McCall B, Smith G, Slinko V, Allen G, Smith I, et al Human Hendra Virus Encephalitis Associated with Equine Outbreak, Australia, 2008. Emerg Infect Dis. 2010; 16: 219–223. doi: 10.3201/eid1602.090552 20113550 10.3201/eid1602.090552 PMC2957996

[8] Rockx B, Winegar R, Freiberg AN. Recent progress in henipavirus research: Molecular biology, genetic diversity, animal models. Antivir Res. 2012; 95: 135–149. doi: 10.1016/j.antiviral.2012.05.008 22643730 10.1016/j.antiviral.2012.05.008

[9] Chong HT, Hossain MJ, Tan CT. Differences in epidemiologic and clinical features of Nipah virus encephalitis between the Malaysian and Bangladesh outbreaks. Neurol Asia. 2008; 13: 23–26.

[10] Luby SP, Gurley ES, Hossain MJ. Transmission of human infection with Nipah virus. Clinical infectious diseases: an official publication of the Infectious Diseases Society of America 2009; 49: 1743–1748. 19886791 10.1086/647951 PMC2784122

[11] Luby SP, Rahman M, Hossain MJ, Blum LS, Husain MM, Gurley E, et al Foodborne transmission of Nipah virus, Bangladesh. Emerg Infect Dis. 2006; 12: 1888–1894. doi: 10.3201/eid1212.060732 17326940 10.3201/eid1212.060732 PMC3291367

[12] Daniels P, Ksiazek T, Eaton BT. Laboratory diagnosis of Nipah and Hendra virus infections. Microbes Infect. 2001; 3(4):289–295. 11334746 10.1016/s1286-4579(01)01382-x

[13] Weingartl HM, Berhane Y, Czub M. Animal models of henipavirus infection: a review. Vet J. 2009; 181: 211–220. doi: 10.1016/j.tvjl.2008.10.016 19084436 10.1016/j.tvjl.2008.10.016

[14] Li M, Embury-Hyatt C, Weingartl HM. Experimental inoculation study indicates swine as a potential host for Hendra virus. Vet Res. 2010; 41: 33 doi: 10.1051/vetres/2010005 20167195 10.1051/vetres/2010005 PMC2826093

[15] Westbury HA, Hooper PT, Brouwer SL, Selleck PW Susceptibility of cats to equine morbillivirus. Aust Vet J. 1996; 74: 132–134. 8894019 10.1111/j.1751-0813.1996.tb14813.x

[16] Hooper PT, Westbury HA, Russell GM. The lesions of experimental equine morbillivirus disease in cats and guinea pigs. Vet Pathol. 1997; 34: 323–329. doi: 10.1177/030098589703400408 9240841 10.1177/030098589703400408

[17] Williamson MM, Hooper PT, Selleck PW, Westbury HA, Slocombe RF.Experimental hendra virus infectionin pregnant guinea-pigs and fruit Bats (Pteropus poliocephalus). J Comp Pathol. 2000; 122: 201–207. doi: 10.1053/jcpa.1999.0364 10684689 10.1053/jcpa.1999.0364

[18] Guillaume V, Wong KT, Looi RY, Georges-Courbot MC, Barrot L, Buckland R, et al Acute Hendra virus infection: Analysis of the pathogenesis and passive antibody protection in the hamster model. Virology 2009; 387: 459–465. doi: 10.1016/j.virol.2009.03.001 19328514 10.1016/j.virol.2009.03.001

[19] Rockx B, Bossart KN, Feldmann F, Geisbert JB, Hickey AC, Brining D, et al A novel model of lethal Hendra virus infection in African green monkeys and the effectiveness of ribavirin treatment. J Virol. 2010; 84: 9831–9839. doi: 10.1128/JVI.01163-10 20660198 10.1128/JVI.01163-10 PMC2937751

[20] Pallister J, Middleton D, Wang LF, Klein R, Haining J, Robinson R, et al A recombinant Hendra virus G glycoprotein-based subunit vaccine protects ferrets from lethal Hendra virus challenge. Vaccine 2011; 29: 5623–5630. doi: 10.1016/j.vaccine.2011.06.015 21689706 10.1016/j.vaccine.2011.06.015 PMC3153950

[21] Eshaghi M, Tan WS, Mohidin TB, Yusoff K. Nipah virus glycoprotein: production in baculovirus and application in diagnosis. Virus Res. 2004; 106: 71–76. doi: 10.1016/j.virusres.2004.06.011 15522449 10.1016/j.virusres.2004.06.011

[22] Eshaghi M, Tan WS, Chin WK, Yusoff K. Purification of the extra-cellular domain of Nipah virus glycoprotein produced in Escherichia coli and possible application in diagnosis. J Biotechnol. 2005; 116: 221–226. doi: 10.1016/j.jbiotec.2004.10.020 15707682 10.1016/j.jbiotec.2004.10.020 PMC7125951

[23] Eshaghi M, Tan WS, Ong ST, Yusoff K. Purification and characterization of Nipah virus nucleocapsid protein produced in insect cells. J Clin Microbiol. 2005; 43: 3172–3177. doi: 10.1128/JCM.43.7.3172-3177.2005 16000431 10.1128/JCM.43.7.3172-3177.2005 PMC1169143

[24] Chen JM, Yu M, Morrissy C, Zhao YG, Meehan G, Sun YX, et al A comparative indirect ELISA for the detection of henipavirus antibodies based on a recombinant nucleocapsid protein expressed in Escherichia coli. J Virol Methods 2006; 136: 273–276. doi: 10.1016/j.jviromet.2006.05.003 16769130 10.1016/j.jviromet.2006.05.003

[25] Yu F, Khairullah NS, Inoue S, Balasubramaniam V, Berendam SJ, The LK, et al Serodiagnosis using recombinant nipah virus nucleocapsid protein expressed in Escherichia coli. J Clin Microbiol. 2006; 44: 3134–3138. doi: 10.1128/JCM.00693-06 16954238 10.1128/JCM.00693-06 PMC1594737

[26] Bossart KN, McEachern JA, Hickey AC, Choudhry V, Dimitrov DS, Eaton BT et al Neutralization assays for differential henipavirus serology using Bio-Plex protein array systems. J Virol Methods 2007; 142: 29–40. doi: 10.1016/j.jviromet.2007.01.003 17292974 10.1016/j.jviromet.2007.01.003

[27] McNabb L, Barr J, Crameri G, Juzva S, Riddell S, Colling A, et al Henipavirus microsphere immuno-assays for detection of antibodies against Hendra virus. J Virol Meth. 2014; 200; 22–28. 10.1016/j.jviromet.2014.01.010 PMC8846554 24508193

[28] Hayman DT, Suu-Ire R, Breed AC, McEachern JA, Wang L, Wood JL et al Evidence of henipavirus infection in West African fruit bats. PLoS One 2008; 3: e2739 doi: 10.1371/journal.pone.0002739 18648649 10.1371/journal.pone.0002739 PMC2453319

[29] Hayman DT, Wang LF, Barr J, Baker KS, Suu-Ire R, Broder CC, et al Antibodies to henipavirus or henipa-like viruses in domestic pigs in Ghana, West Africa. PLoS One 2011; 6: e25256 doi: 10.1371/journal.pone.0025256 21966471 10.1371/journal.pone.0025256 PMC3178620

[30] Kirkland PD, Gabor M, Poe I, Neale K, Chaffey K, Finlaison DS, et al Hendra Virus Infection in Dog, Australia, 2013. Emerg Infect Dis. 2015; 21: 2182–2185. doi: 10.3201/eid2112.151324 26583697 10.3201/eid2112.151324 PMC4672422

[31] Chowdhury SS, Khan U, Crameri G, Epstein JH, Broder CC, Islam A, et al Serological evidence of henipavirus exposure in cattle, goats and pigs in Bangladesh. PLoS Negl Trop Dis. 2014; 8, e3302 doi: 10.1371/journal.pntd.0003302 25412358 10.1371/journal.pntd.0003302 PMC4238985

[32] Mills JN, Alim AN, Bunning ML, Lee OB, Wagoner KD, Amman BR, et al Nipah virus infection in dogs, Malaysia, 1999. Emerg Infect Dis. 2009; 15: 950–952. doi: 10.3201/eid1506.080453 19523300 10.3201/eid1506.080453 PMC2727347

[33] Wang LF, Harcourt BH, Yu M, Tamin A, Rota PA, Bellini WJ, et al Molecular biology of Hendra and Nipah viruses. Microbes Infect. 2001; 3: 279–287. 11334745 10.1016/s1286-4579(01)01381-8

[34] Towbin H, Staehelin T, Gordon J. Electrophoretic transfer of proteins from polyacrylamide gels to nitrocellulose sheets: procedure and some applications. PNAS 1979; 76: 4350–4354. 388439 10.1073/pnas.76.9.4350 PMC411572

[35] Berhane YJD, Berry JD, Ranadheera C, Marszal P, Nicolas B, Yuan X, et al Production and characterization of monoclonal antibodies against binary ethylenimine inactivated Nipah virus. J Virol Methods 2006; 132: 59–68. doi: 10.1016/j.jviromet.2005.09.005 16226320 10.1016/j.jviromet.2005.09.005

[36] Fischer K, Dos Reis VP, Finke S, Sauerhering L, Stroh E, Karger A et al Expression, characterisation and antigenicity of a truncated Hendra virus attachment protein expressed in the protozoan host Leishmania tarentolae. J Virol Methods 2016; 228: 48–54. doi: 10.1016/j.jviromet.2015.11.006 26585033 10.1016/j.jviromet.2015.11.006

[37] Kohler G, Milstein C. Continuous cultures of fused cells secreting antibody of predefined specificity. Nature 1975; 256: 495–497. 1172191 10.1038/256495a0

[38] Pontecorvo G. Production of mammalian somatic cell hybrids by means of polyethylene glycol treatment. Somatic Cell Genet. 1975; 1: 397–400. 1242069 10.1007/BF01538671

[39] Weingartl HM, Berhane Y, Caswell JL, Loosmore S, Audonnet JC, Roth JA et al Recombinant nipah virus vaccines protect pigs against challenge. J Virol. 2006; 80(16): 7929–7938. doi: 10.1128/JVI.00263-06 16873250 10.1128/JVI.00263-06 PMC1563797

[40] Weingartl H, Czub S, Copps J, Berhane Y, Middleton D, Marszal P et al Invasion of the central nervous system in a porcine host by nipah virus. J Virol. 2005;79(12): 7528–7534. doi: 10.1128/JVI.79.12.7528-7534.2005 15919907 10.1128/JVI.79.12.7528-7534.2005 PMC1143674

[41] Berhane Y, Weingartl HM, Lopez J, Neufeld J, Czub S, Embury-Hyatt C. Bacterial infections in pigs experimentally infected with Nipah virus. Transbound Emerg Dis. 2008; 55(3–4): 165–174. doi: 10.1111/j.1865-1682.2008.01021.x 18405339 10.1111/j.1865-1682.2008.01021.x

[42] Chua KB. Nipah virus outbreak in Malaysia. J Clin Virol. 2003; 26: 265–275. 12637075 10.1016/s1386-6532(02)00268-8

[43] Lo MK, Rota PA. Molecular Virology of the Henipaviruses. Curr Top Microbiol. 2012; 359: 41–58. 10.1007/82_2012_211 22552699

[44] Hughes K. Focus on: Hendra virus in Australia. Vet Rec. 2014; 175: 533–534. doi: 10.1136/vr.g6836 25431383 10.1136/vr.g6836

[45] Middleton D. Hendra virus. Vet Clin North Am Equine Pract. 2014; 30: 579–589. doi: 10.1016/j.cveq.2014.08.004 25281398 10.1016/j.cveq.2014.08.004 PMC4252762

[46] Breitling R, Klingner S, Callewaert N, Pietrucha R, Geyer A, Ehrlich G, et al Non-pathogenic trypanosomatid protozoa as a platform for protein research and production. Protein Expr Purif. 2002; 25: 209–218. 12135552 10.1016/s1046-5928(02)00001-3

[47] Grzyb K, Czarnota A, Brzozowska A, Cieślik A, Rąbalski Ł, Tyborowska J, et al Immunogenicity and functional characterization of Leishmania-derived hepatitis C virus envelope glycoprotein complex. Sci Rep. 2016; 6: 30627 doi: 10.1038/srep30627 27481352 10.1038/srep30627 PMC4969751

[48] Pion C, Courtois V, Husson S, Bernard MC, Nicolai MC, Talaga P, et al Characterization and immunogenicity in mice of recombinant influenza haemagglutinins produced in Leishmania tarentolae. Vaccine 2014; 32(43): 5570–6. doi: 10.1016/j.vaccine.2014.07.092 25131728 10.1016/j.vaccine.2014.07.092

[49] Baechlein C, Meemken D, Pezzoni G, Engemann C, Grummer B. Expression of a truncated hepatitis E virus capsid protein in the protozoan organism Leishmania tarentolae and its application in a serological assay. J Virol Methods 2013; 193(1): 238–43. doi: 10.1016/j.jviromet.2013.05.018 23747546 10.1016/j.jviromet.2013.05.018

[50] Basile G, Peticca M. Recombinant protein expression in Leishmania tarentolae. Mol Biotechnol. 2009; 43(3):273–8. doi: 10.1007/s12033-009-9213-5 19779853 10.1007/s12033-009-9213-5

[51] Pearce LA, Yu M, Waddington LJ, Barr JA, Scoble JA, Crameri GS et al Structural characterization by transmission electron microscopy and immunoreactivity of recombinant Hendra virus nucleocapsid protein expressed and purified from Escherichia coli. Protein Expr Purif. 2015; 116: 19–29. doi: 10.1016/j.pep.2015.07.008 26196500 10.1016/j.pep.2015.07.008 PMC7129954

[52] Tan WS, Ong ST, Eshaghi M, Foo SS, Yusoff K. Solubility, immunogenicity and physical properties of the nucleocapsid protein of Nipah virus produced in Escherichia coli. J Med Virol. 2004; 73(1):105–12. doi: 10.1002/jmv.20052 15042656 10.1002/jmv.20052

[53] Bossart KN, Crameri G, Dimitrov AS, Mungall BA, Feng YR, Patch JR, et al Receptor binding, fusion inhibition, and induction of cross-reactive neutralizing antibodies by a soluble G glycoprotein of Hendra virus. J Virol. 2005; 79: 6690–6702. doi: 10.1128/JVI.79.11.6690-6702.2005 15890907 10.1128/JVI.79.11.6690-6702.2005 PMC1112112

[54] Negrete OA, Levroney EL, Aguilar HC, Bertolotti-Ciarlet A, Nazarian R, Tajyar S et al EphrinB2 is the entry receptor for Nipah virus, an emergent deadly paramyxovirus. Nature 2005; 436(7049): 401–405. doi: 10.1038/nature03838 16007075 10.1038/nature03838

[55] Negrete OA, Wolf MC, Aguilar HC, Enterlein S, Wang W, Mühlberger E et al Two key residues in ephrinB3 are critical for its use as an alternative receptor for Nipah virus. PLoS Pathog. 2006; 2(2):e7 doi: 10.1371/journal.ppat.0020007 16477309 10.1371/journal.ppat.0020007 PMC1361355

[56] Harcourt BH, Tamin A, Ksiazek TG, Rollin PE, Anderson LJ, Bellini WJ, et al Molecular characterization of Nipah virus, a newly emergent paramyxovirus. Virology 2000; 271: 334–349. doi: 10.1006/viro.2000.0340 10860887 10.1006/viro.2000.0340

[57] Marsh GA, de Jong C, Barr JA, Tachedjian M, Smith C, Middleton D, et al Cedar Virus: A Novel Henipavirus Isolated from Australian Bats. PLoS Pathog. 2012; 8. 10.1371/journal.ppat.1002836 PMC3410871 22879820

[58] de Araujo J, Lo MK, Tamin A, Ometto TL, Thomazelli LM, Nardi MS, et al Antibodies Against Henipa-Like Viruses in Brazilian Bats. Vector borne Zoonotic Dis. 2017; 17(4):271–274. doi: 10.1089/vbz.2016.2051 28103156 10.1089/vbz.2016.2051

[59] Kulkarni DD, Venkatesh G, Tosh C, Patel P, Mashoria A, Gupta V, et al Development and Evaluation of Recombinant Nucleocapsid Protein Based Diagnostic ELISA for Detection of Nipah Virus Infection in Pigs. J Immunoassay Immunochem. 2016; 37(2):154–166. doi: 10.1080/15321819.2015.1074922 26327601 10.1080/15321819.2015.1074922

[60] Mungall BA, Middleton D, Crameri G, Bingham J, Halpin K, Russell G, et al Feline model of acute nipah virus infection and protection with a soluble glycoprotein-based subunit vaccine. J Virol. 2006; 80: 12293–12302. doi: 10.1128/JVI.01619-06 17005664 10.1128/JVI.01619-06 PMC1676295

[61] Pallister JA, Klein R, Arkinstall R, Haining J, Long F, White JR, et al Vaccination of ferrets with a recombinant G glycoprotein subunit vaccine provides protection against Nipah virus disease for over 12 months. Virol J. 2013; 10: 237 doi: 10.1186/1743-422X-10-237 23867060 10.1186/1743-422X-10-237 PMC3718761

[62] McEachern JA, Bingham J, Crameri G, Green DJ, Hancock TJ, Middleton D, et al A recombinant subunit vaccine formulation protects against lethal Nipah virus challenge in cats. Vaccine 2008; 26: 3842–3852. doi: 10.1016/j.vaccine.2008.05.016 18556094 10.1016/j.vaccine.2008.05.016 PMC6186147

[63] Bossart KN, Rockx B, Feldmann F, Brining D, Scott D, LaCasse R, et al A Hendra virus G glycoprotein subunit vaccine protects African green monkeys from Nipah virus challenge. Science Translat Medicine. 2012, 4: 146ra107. 10.1126/scitranslmed.3004241 PMC3516289 22875827

[64] Pickering BS, Hardham JM, Smith G, Weingartl ET, Dominowski PJ, Foss DL, et al Protection against henipaviruses in swine requires both, cell-mediated and humoral immune response. Vaccine 2016; 34: 4777–4786. doi: 10.1016/j.vaccine.2016.08.028 27544586 10.1016/j.vaccine.2016.08.028 PMC6161494

[65] Drexler JF, Corman VM, Gloza-Rausch F, Seebens A, Annan A, Ipsen A, et al Henipavirus RNA in African Bats. PLoS One 2009; 4. 10.1371/journal.pone.0006367 PMC2712088 19636378

[66] Drexler JF, Corman VM, Muller MA, Maganga GD, Vallo P, Binger T, et al Bats host major mammalian paramyxoviruses. Nature Commun. 2012; 3: 796. 22531181 10.1038/ncomms1796 PMC3343228

[67] Iehle C, Razafitrimo G, Razainirina J, Andriaholinirina N, Goodman SM, Faure C, et al Henipavirus and Tioman virus antibodies in pteropodid bats, Madagascar. Emerg Infect Dis. 2007; 13: 159–161. doi: 10.3201/eid1301.060791 17370536 10.3201/eid1301.060791 PMC2725826

[68] Pernet O, Schneider BS, Beaty SM, LeBreton M, Yun TE, Park A. Evidence for henipavirus spillover into human populations in Africa. Nature Comm. 2014; 5: 5342. 10.1038/ncomms6342 PMC4237230 25405640

