# Supplementary figures and images for: Indirect ELISA based on Hendra and Nipah virus proteins for the detection of henipavirus specific antibodies in pigs
Source: PLoS One. 2018 Apr 30;13(4):e0194385. doi: 10.1371/journal.pone.0194385 (PMC5927399; doi:10.1371/journal.pone.0194385)

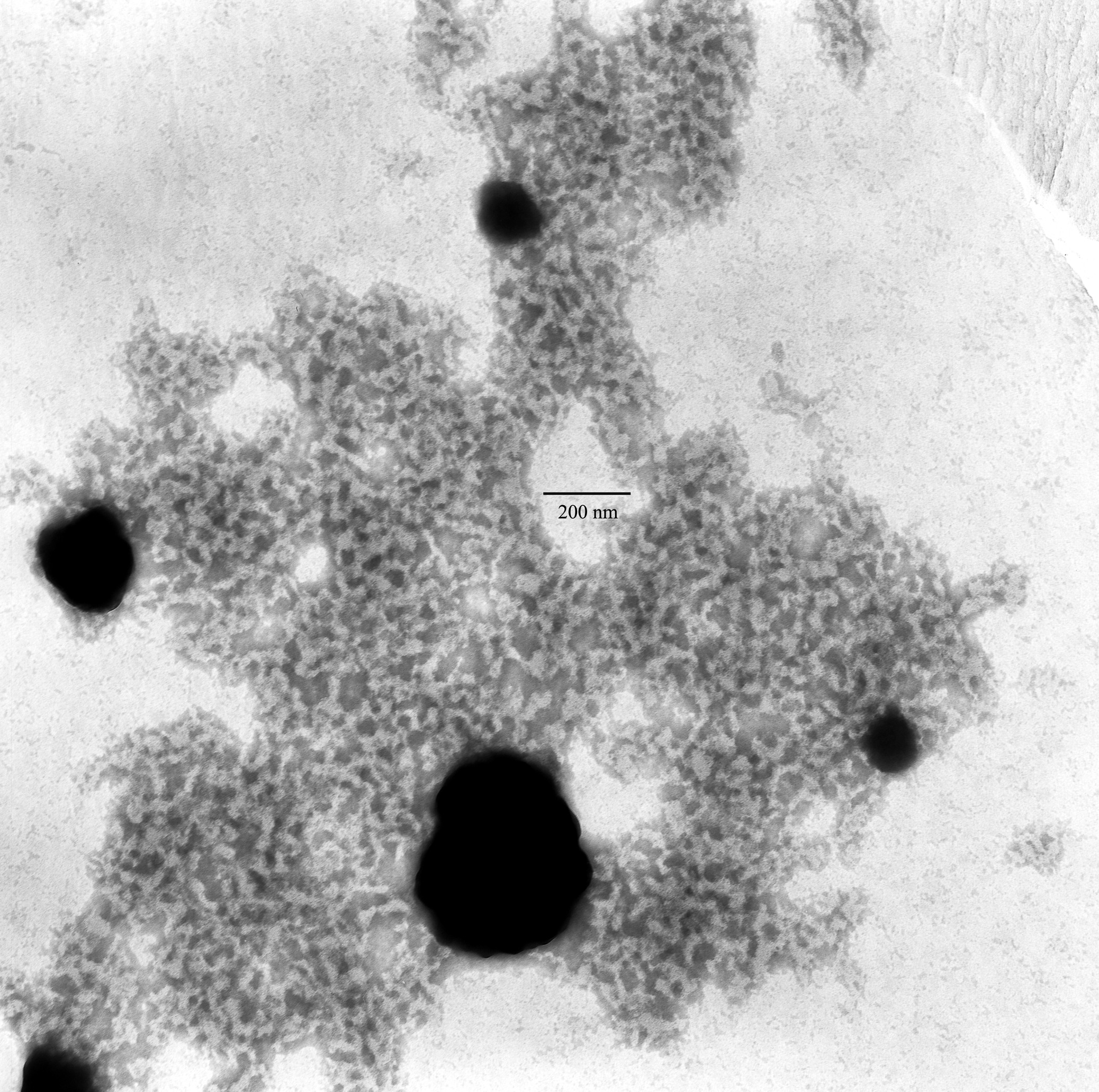

Supplement: S1 Fig — The specimen grids were examined in a Philips CM 120 transmission electron microscope, operating at an accelerating voltage of 80 kV. Micrographs were taken between 28,000X–45,000X using Kodak Electron Microscope Film 4489. The negatives were scanned using an Epson Perfection 3200 photo scanner and enlarged 2.5X. (TIF) [file pone.0194385.s001.TIF]

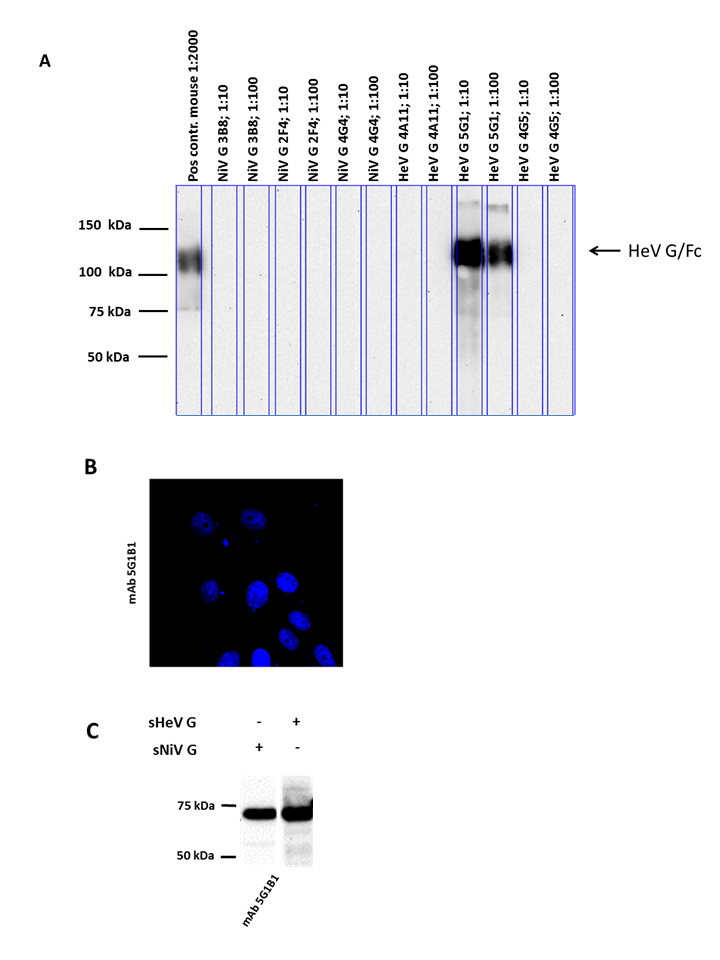

Supplement: S2 Fig — A. Western blot analysis of 5G1B1 reactivity against commercially obtained HeV G/Fc (Sino Biologicals). The monoclonal antibody hybridoma supernatant 5G1B1 was utilized in a dilution of 1:10 and 1:100. Other hybridoma supernatants were tested but did not reveal positive signal in Western blot. B. Immunofluorescence analysis of monoclonal antibody 5G1B1 reactivity against Mock-transfected Vero76 cells. Vero 76 cells were transfected with the pCAGGS plasmid. For immunostaining, the newly generated cross-reactive monoclonal antibody 5G1B1 was used followed by mouse specific Alexa-Fluor 488-labeled secondary antibodies. Nuclei were stained with Hoechst. Fluorescence was visualized by a DMI7 live cell microscope (Leica), magnification 630 x. C. Western blot analysis of 5G1B1 reactivity against Leishmania-derived sHeV or NiV G. The monoclonal antibody hybridoma supernatant 5G1B1 was utilized in a dilution of 1:100. (TIF) [file pone.0194385.s002.TIF]

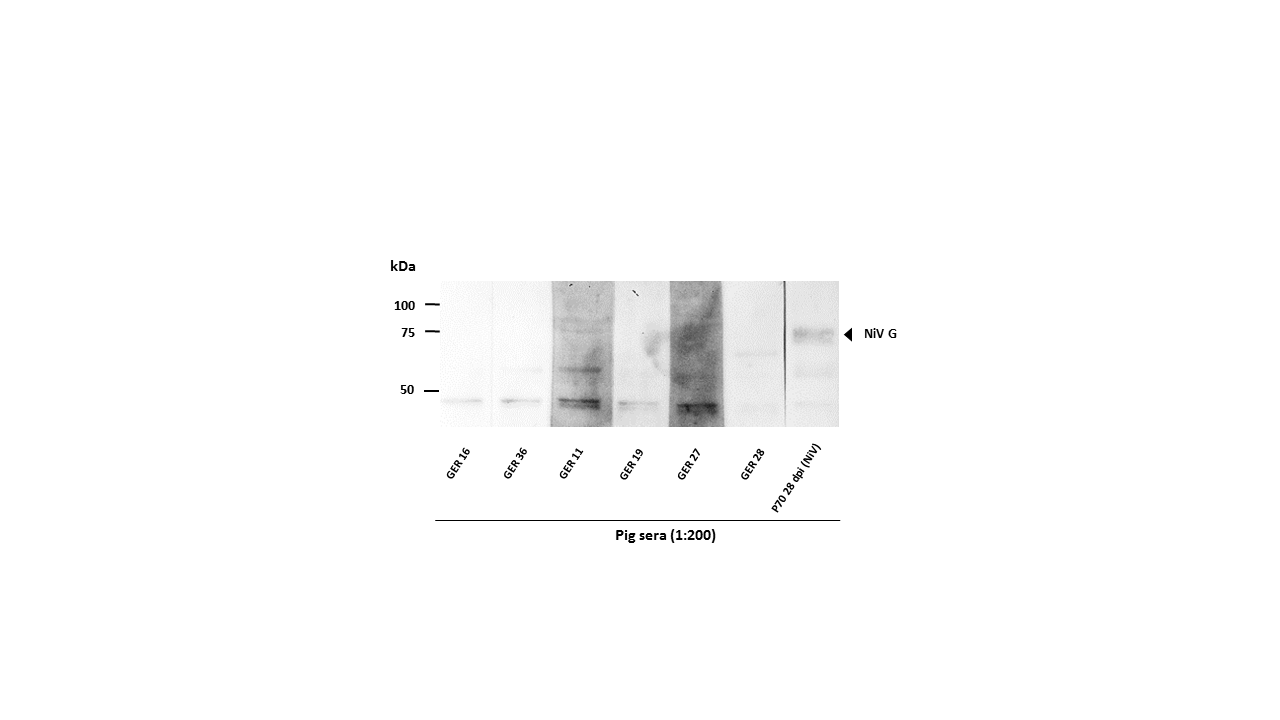

Supplement: S3 Fig — Serum sample from a NiV infected pig was collected at 7 dpi served as a positive control. Six German pig sera that exceeded the calculated cut-off values in or or both G based ELISAs were tested for reactivity in immunoblot analysis. All sera were diluted as indicated. The monoclonal antibody 5G1B1 was utilized in a dilution of 1:100. (TIF) [file pone.0194385.s003.TIF]
